# Murine T-Lymphomas Corresponding to the Immature CD4^-^8^+^ Thymocyte Subset

**DOI:** 10.1155/1991/98963

**Published:** 1991

**Authors:** Ellen R. Richie, Brenda B. Mcentire, Lezlee Coghlan, Martin Poenie

**Affiliations:** 1 The University of Texas M. D. Anderson Cancer Center Science Park-Research Division P.O. Box 389 Smithville Texas 78957 USA; 2 The Department of Zoology The University of Texas at Austin Austin Texas 78712 USA

## Abstract

N-methyl-N-nitrosourea induces murine CD4^-^8^+^ T-lymphomas that express high levels of J11d and low levels of CD5 antigens, a phenotype characteristic of immature CD4^-^8^+^ thymocytes. This assignment is supported by the fact that CD4^-^8^+^ lymphoma cell lines acquire CD4 expression after intrathymic (i.t.) transfer, a finding consistent with the established precursor potential of the normal immature CD4^-^8^+^ subset. CD4^+^8^+^ lymphomas recovered after i.t. transfer maintain a CD4^+^8^+^ phenotype in long-term culture. Northern blot analyses reveal that CD4 expression is regulated at the transcriptional level in immature CD4^-^8^+^ and CD4^+^8^+^ cell lines. CD4^-^8^+^ lymphomas express low levels of functional CD3/TCR complexes that mediate intracellular Ca2^+^ mobilization in response to CD3 or α/β-TCR monoclonal antibody. These data suggest that the immature CD4^-^8^+^ subset contains cells capable of undergoing TCR-mediated signaling and selection events. In contrast to normal immature CD4^-^8^+^ cells, which comprise a heterogeneous and transient subset, the CD4^-^8^+^ lymphoma lines provide stable, monoclonal models of the immature CD4^-^8^+^ stage of thymocyte development.


*Developmental Immunology*, 1991, Vol. 1, pp. 255–263
Reprints available directly from the publisher
Photocopying permitted by license only

© 1991 Harwood Academic Publishers GmbH
Printed in the United Kingdom

### Murine T-Lymphomas Corresponding to the Immature $CD4^-8^+$ Thymocyte Subset

ELLEN R. RICHIE,*† BRENDA B. McENTIRE,† LEZLEE COGHLAN,† and MARTIN POENIE‡

†*The University of Texas M. D. Anderson Cancer Center, Science Park-Research Division, P.O. Box 389, Smithville, Texas 78957*
‡*The Department of Zoology, The University of Texas at Austin, Austin, Texas 78712*

N-methyl-N-nitrosourea induces murine $CD4^-8^+$ T-lymphomas that express high levels of J11d and low levels of CD5 antigens, a phenotype characteristic of immature $CD4^-8^+$ thymocytes. This assignment is supported by the fact that $CD4^-8^+$ lymphoma cell lines acquire CD4 expression after intrathymic (i.t.) transfer, a finding consistent with the established precursor potential of the normal immature $CD4^-8^+$ subset. $CD4^+8^+$ lymphomas recovered after i.t. transfer maintain a $CD4^+8^+$ phenotype in long-term culture. Northern blot analyses reveal that CD4 expression is regulated at the transcriptional level in immature $CD4^-8^+$ and $CD4^+8^+$ cell lines. $CD4^-8^+$ lymphomas express low levels of functional CD3/TCR complexes that mediate intracellular $Ca2^+$ mobilization in response to CD3 or α/β-TCR monoclonal antibody. These data suggest that the immature $CD4^-8^+$ subset contains cells capable of undergoing TCR-mediated signaling and selection events. In contrast to normal immature $CD4^-8^+$ cells, which comprise a heterogeneous and transient subset, the $CD4^-8^+$ lymphoma lines provide stable, monoclonal models of the immature $CD4^-8^+$ stage of thymocyte development.

KEYWORDS: T-cell development, T-lymphoma, thymocyte subsets.

### INTRODUCTION

T-lymphocytes mature within the thymus, which provides the appropriate cellular and humoral microenvironment to induce differentiation of immature precursors into functional T-cells (Adkins et al., 1986). Normal adult thymocytes are classically grouped into four populations based on expression of CD4 and CD8 accessory molecules. The $CD4^-8^-$ subset contains intrathymic precursors that give rise to other thymocyte subsets in irradiated hosts or in organ culture (Fowlkes et al., 1985). The immature $CD4^+8^+$ subset constitutes the major thymocyte subset. Most $CD4^+8^+$ cells express low levels of clonotypic α/β-TCR and are destined to die in situ presumably as a result of negative selection or failure to undergo positive selection (McPhee et al., 1979; Havran et al., 1987; Richie et al., 1988). $CD4^+8^-$ and $CD4^-8^+$ medullary cells express high levels of α/β-TCRs and are functionally, as well as phenotypically, similar to thymic emigrants and peripheral T cells (Ceredig et al., 1982; Scollay et al., 1984).

Although the classification scheme based on CD4 and CD8 expression has been useful in studying the relationships among thymocyte subsets, it is increasingly evident that considerable heterogeneity exists within each subset. For example, the $CD4^-8^-$ compartment contains cells that differ in antigen expression as well as in precursor potential (Adkins et al., 1986). Recent studies have demonstrated that the $CD4^-8^+$ subset also is heterogeneous in anatomical location, α/β-TCR expression and functional potential. In addition to mature medullary $CD4^-8^+$ cells, a distinct subset of functionally immature $CD4^-8^+$ cells in the adult and neonatal thymic cortex is characterized by undetectable or low levels of CD3 expression and high levels of heat-stable antigen (HSA) expression (Crispe and Bevan, 1987; Ceredig, 1988). This subset represents a transient population of cycling thymocytes that is intermediate between $CD4^-8^-$ and $CD4^+8^+$ maturation stages (Penit and Vasseur, 1988). Acquisition of CD4 expression occurs on immature $CD4^-8^+$ cells after intrathymic transfer or in vitro

*Corresponding author.

incubation (MacDonald et al., 1988; Guidos et al., 1989).

Due to the transient nature and heterogeneous composition of the immature CD4⁻8⁺ subset in normal thymus, it has not been possible to investigate the acquisition of CD4 expression in a clonal population, or to define the functional potential of those CD4⁻8⁺ cells that express low levels of CD3/TCR complex. To address these issues, we developed clonal cell lines from thymic lymphomas that represent the immature $CD4^-8^+CD3^{lo}$ thymocyte subset. In a previous study, we reported that the majority of N-methyl-N-nitrosourea-(MNU)induced thymic lymphomas in AKR mice express a $CD4^-8^+$ phenotype (Richie, 1988). These lymphomas also express ThB, a differentiation antigen present on immature but not on mature T-cells (Yotoku et al., 1976), suggesting that they correspond to an immature differentiation stage. The present report describes the phenotypic and functional properties of $CD4^-8^+$ MNU-induced lymphomas established in culture as continuous cell lines. The data support the assignment of these lines to the immature $CD4^-8^+$ subset and demonstrate their precursor potential. In addition, transcriptional control of CD4 expression and signal transduction via the CD3/TCR complex are assessed prior to and after i.t. transfer.

### RESULTS

### Phenotypic Analysis of $CD4^-8^+$ Lymphoma Cell Lines

Mature thymocytes express either CD4 or CD8 molecules. Recently, however, a minor subset of $CD4^-8^+$ thymocytes has been described that displays functional and phenotypic properties characteristic of immature cells (Crispe and Bevan, 1987; Ceredig, 1988; MacDonald et al., 1988; Penit and Vasseur, 1988; Guidos et al., 1989). MNU-induced lymphomas obtained from AKR/J mice generally express a $CD4^-8^+$ antigen profile (Richie, 1988). To determine if these lymphomas represent the mature or immature CD8 single-positive (SP) subset, two MNU-induced lymphoma cell lines (829M and 802M) were further characterized. The data in Fig. 1 are consistent with assignment of an immature status for both cell lines since they express high levels of

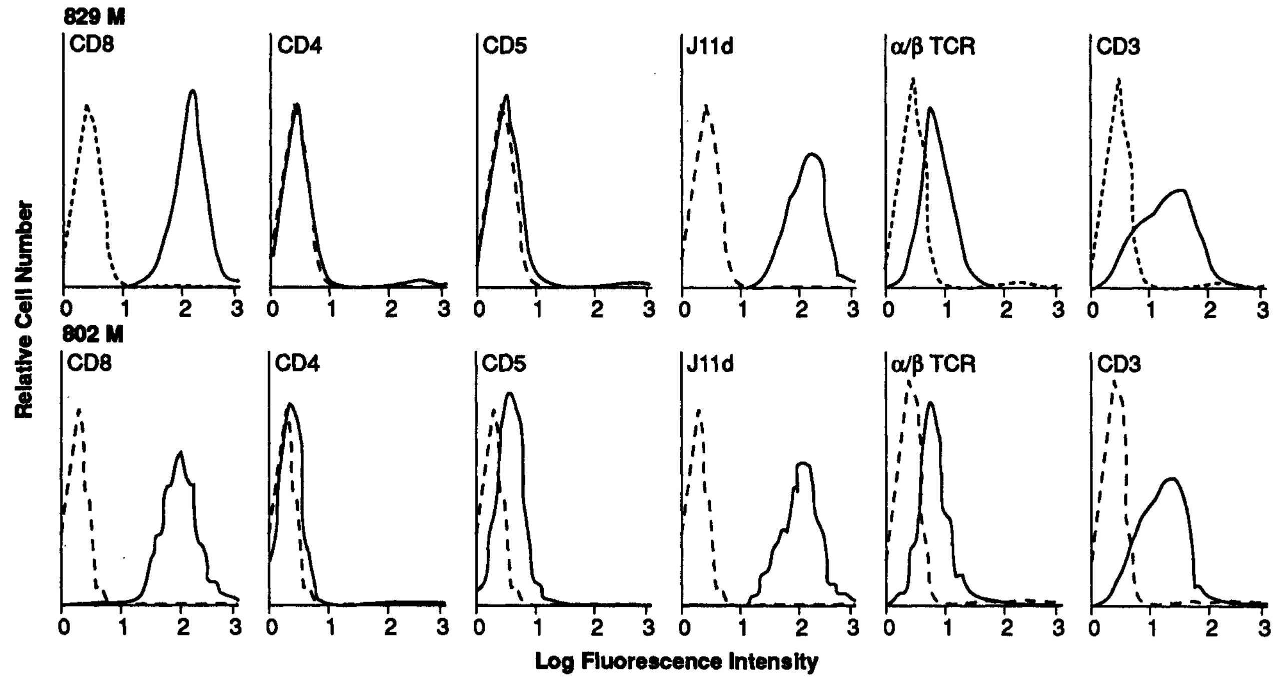

FIGURE 1. Differentiation antigen phenotype of 829M (upper panel) and 802M (lower panel) lymphoma lines. The dashed line in each histogram shows the profile for cells stained only with an appropriate second-step reagent.

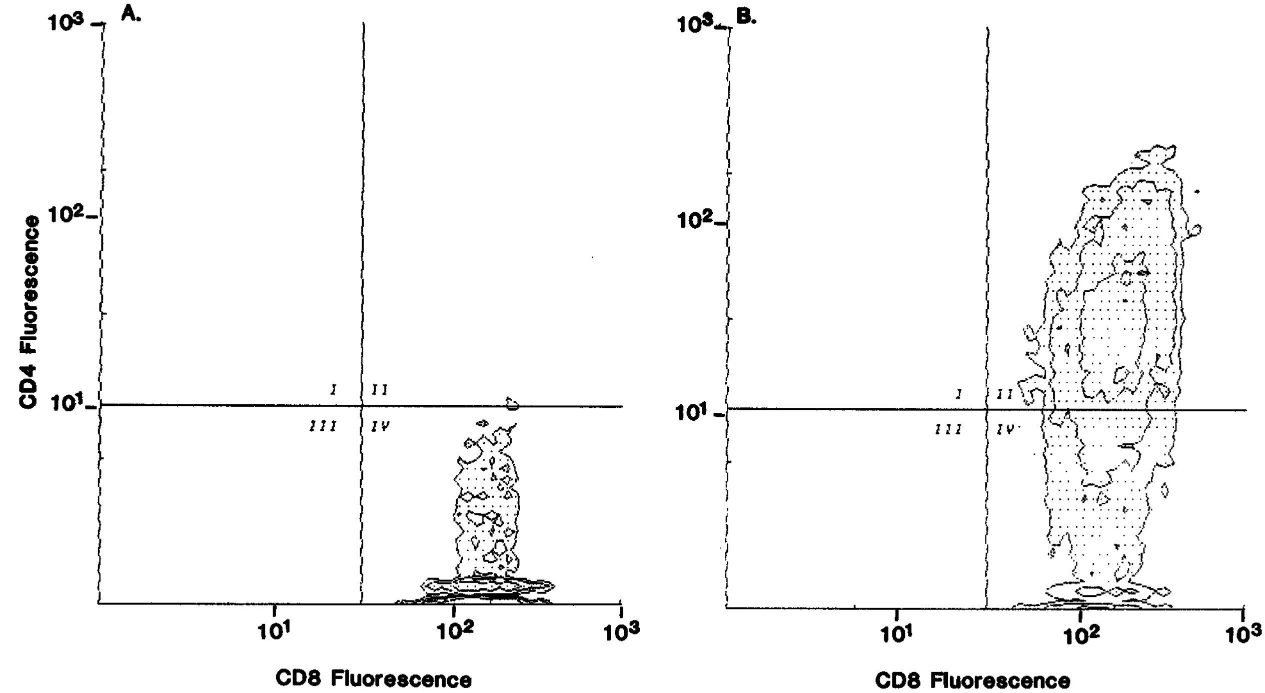

FIGURE 2. CD4 and CD8 expression on 829M cells obtained (A) prior to and (B) after i.t. transfer. Contour plots from two-color immunofluorescence analysis of cells incubated with FITC-conjugated anti-CD8 (horizontal axis) and PE-conjugated anti-CD4 (vertical axis).

HSA (detected by mAb J11d), undetectable or low levels of CD5 antigen, and low or moderate levels of CD3 and α/β-TCR.

### Intrathymic Transfer of CD4⁻8⁺ Cell Lines

Immature CD4⁻8⁺ thymocytes are thought to represent a transient, intermediary subset between the CD4⁻8⁻ and CD4⁺8⁺ developmental stages (Penit and Vasseur, 1988). Previous studies demonstrating the generation of double-positive (DP) cells from immature SP progenitors were carried out with polyclonal normal thymocytes (MacDonald et al., 1988; Nikolic-Zugic and Bevan, 1988; Guidos et al., 1989). To investigate the developmental potential of monoclonal immature CD4⁻8⁺ cells, the 829M and 802M cell lines (Thy1.1⁺) were transferred by i.t. injection into irradiated and syngeneic bone marrow reconstituted AKR/C (Thy1.2⁺) mice. Intrathymic proliferation of lymphoma cells was apparent from the enlarged recipient thymuses and recovery of >90% Thy 1.1⁺ cells. As shown in Fig. 2, the vast majority (93%) of 829M cells recovered after i.t. injection displayed a CD4⁺8⁺ DP phenotype. CD4 expression was also acquired on the 802M cell lines after i.t. transfer, although in this case

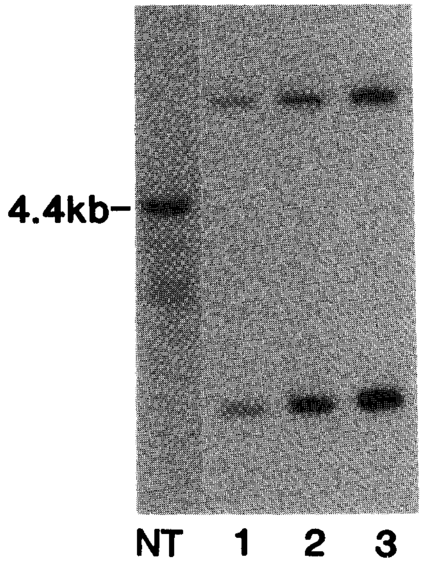

FIGURE 3. Comparison of Jβ2 gene rearrangements in normal thymus (NT), 829M, and 7-829M cells. DNA was digested with HindIII and Southern blots were hybridized with a probe specific for the Jβ2 gene segment (Malissen et al., 1984). Lane 1, 829M DNA; lane 2, DNA from 829M cells recovered after i.t. transfer; lane 3, 7-829M DNA.

fewer (65%) of the recovered cells expressed a DP phenotype (data not shown). To investigate the clonal relationship of the injected SP and recovered DP populations, Southern blot analyses of

TCR β-chain gene rearrangements were carried out. Figure 3 shows that the pattern of Jβ2 gene rearrangements in the $CD4^+8^+$ 829M cells recovered after i.t. transfer was identical to that in the original $CD4^-8^+$ cell line. These results show that the precursor activity described for heterogeneous populations of normal $CD4^-8^+$ thymocytes is also a property of the monoclonal 829M and 802M lymphoma lines.

### Stable Expression of CD4 on 829M Cells Obtained after Intrathymic Transfer

To determine if the acquisition of CD4 expression was a stable phenomenon, $CD4^+8^+$ lymphoma cells recovered after i.t. transfer of the 829M line were established in vitro as a cell line designated 7-829M. The clonal pattern of Jβ2 gene rearrangements in this cell line was identical to that found in the parental 829M cells (Fig. 3). Analyses of CD4 expression at various intervals revealed that the 7-829M cells continued to express CD4 during several months of continuous culture (Fig. 4).

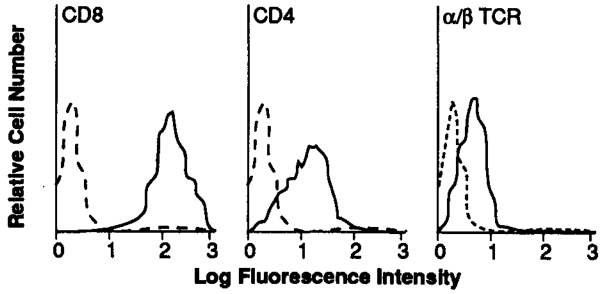

FIGURE 4. Differentiation antigen phenotype of the 7-829M lymphoma line. The cells were maintained in continuous culture for several months when indirect immunofluorescence analyses were performed.

### Northern Blot Analyses of CD4 Transcripts

CD4 gene expression of 829M cells before and after i.t. transfer was assessed by Northern blot analysis. Cytoplasmic or poly($A^+$) RNA was obtained from the CD4 negative 829M cell line, the CD4 positive 829M lymphoma cells recovered after i.t. injection, and the CD4 positive 7-829M cell line. Figure 5 shows that the 829M cell line did not contain detectable levels of CD4 transcripts. In contrast, the DP cells recovered after i.t. transfer and the 7-829M cell line contained readily detectable CD4 mRNA. These data suggest that the failure of 829M cells to express cell-surface CD4 antigen is not due to masking of the antigen or defective translation, but rather to negative transcriptional regulation or an alteration in CD4 mRNA stability. In either case, exposure of the CD4 negative cell line to the thymic microenvironment positively influences up-regulation of steady-state CD4 transcript levels.

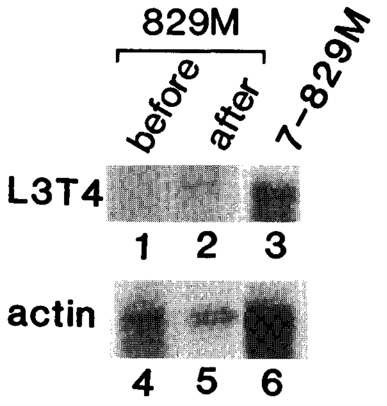

FIGURE 5. Comparison of steady-state levels of CD4 mRNA in 829M and 7-829M cell lines. Lane 1, poly$(A)^+$ RNA from the 829M cell line; lane 2, total cellular RNA from 829M cells recovered after i.t. transfer; lane 3, poly$(A)^+$ RNA from the 7-829M cell line. Northern blots were hybridized with a CD4-specific hybridization probe (Littman and Gettner, 1987).

### Mobilization of Intracellular $Ca^{2+}$ in Response to CD3/TCR Cross-Linking

Previous studies have shown that anti-CD3 mAb induces a positive but marginal increase in intracellular $Ca^{2+}$ mobilization in the $CD4^+8^+$ thymocyte subset (Havran et al., 1987). Recent comparative analysis of signaling in normal thymocytes demonstrated that the $Ca^{2+}$ transient induced by anti-α/β-TCR is reduced in comparison with that generated by anti-CD3 (Finkel, Cambier et al., 1989; Finkel, Marrack et al., 1989). It was suggested that this disparity reflects inefficient coupling of CD3 and α/β-TCR in a subpopulation of immature thymocytes. To compare the $Ca^{2+}$ mobilization response induced by CD3 and α/β-TCR mAb and determine if altered signaling via the CD3/TCR complex accompanied conversion to a DP phenotype, the 829M and 7-829M cells were loaded with the $Ca^{2+}$-sensitive indicator Indo-1. As shown in Fig. 6, cross-linked anti-CD3 stimulated a large $Ca^{2+}$ transient that rapidly declined in both cell lines. The $Ca^{2+}$ response stimulated

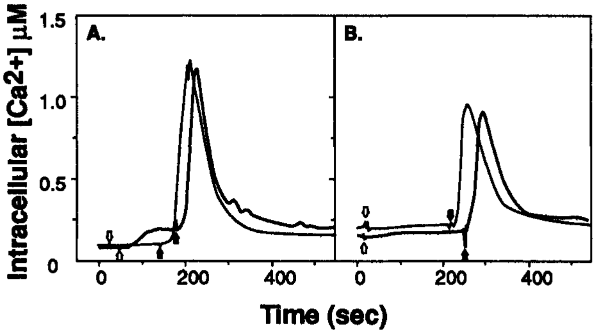

FIGURE 6. Increase in intracellular free [$Ca^{2+}$] levels in 829M (panel A) and 7-829M (panel B) cells following activation with anti-CD3 or anti-α/βTCR (bold line) mAb. The primary mAb was added at initiation (open arrows) and cross-linking was achieved by subsequent addition of goat antihamster Ig (closed arrows).

by cross-linked anti-α/β-TCR was similar in magnitude and kinetics to that induced by anti-CD3 activation, indicating the presence of a functionally coupled receptor complex. After the maximum $Ca^{2+}$ flux was triggered by cross-linked mAb, the level of intracellular $Ca^{2+}$ remained above base line at approximately 0.25 µM for an extended period. Interestingly, anti-α/β-TCR, but not anti-CD3 mAb, stimulated a small but persistent increase in intracellular $Ca^{2+}$ concentration in the absence of cross-linking reagent in the 829M cells.

### DISCUSSION

Investigations of normal thymocyte subsets involve heterogeneous populations even when the cells are selectively enriched for a particular antigenic profile. The establishment of lymphoma cell lines that express an immature $CD4^-8^+$ phenotype provided an opportunity to investigate this unique subset using monoclonal populations. The conclusion that the MNU-induced lymphoma lines represent the immature $CD4^-8^+$ subset is based, in part, on their low-level expression of CD5 and high-level expression of J11d antigens. In addition, histological analyses of thymic sections from MNU-treated mice reveal that neoplastic foci develop in the thymic cortex rather than in the medulla, as might be expected if a mature $CD4^-8^+$ clone underwent neoplastic expansion (Stettner et al., in press). The most compelling data suggesting that these lymphomas are neoplastic counterparts of the immature $CD4^-8^+$ subset are their differentiation potentials. Intrathymic transfer of the 829M and 802M cell lines resulted in acquisition of cell-surface CD4 expression. Since these studies involved monoclonal populations, it was possible to verify, by TCR β-chain gene-rearrangement patterns, that the $CD4^+8^+$ cells recovered after i.t. transfer were clonal descendants of $CD4^-8^+$ precursors. The 7-829M cell line established after intrathymic transfer remained CD4 positive for several months, indicating that sustained contact with the thymic microenvironment is not required to maintain CD4 expression on these cells.

There is some disagreement regarding CD3 expression on the immature $CD4^-8^+$ subset in normal thymus. One study failed to find $CD3^+$ cells in the $HSA^{hi}$ subset of isolated $CD4^-8^+$ normal thymocytes (Shortman et al., 1988). On the other hand, another group reported that failure to express CD3 is not a universal feature of all immature $CD4^-8^+$ cells (MacDonald et al., 1988). Rather, their data are consistent with a low frequency of receptor-bearing immature $CD4^-8^+$ cells. Low levels of receptor also were detected on 50% of immature $CD4^-8^+$ rat thymocytes (Hunig, 1988). Since this study was performed in rats, the data may not be strictly comparable to thymocyte subsets of the mouse. Nevertheless, the results support the notion that the CD3/TCR complex is expressed on a fraction of the immature $CD4^-8^+$ subset. Thus, expression of the CD3/TCR complex on the 829M and 802M cell lines is consistent with a phenotypically similar counterpart representing a transitional stage in normal T-cell differentiation.

The CD3 and α/β-TCR molecules expressed on the $CD4^-8^+$ 829M and $CD4^+8^+$ 7-829M cells appear to be functionally coupled insofar as cross-linked mAb to either component triggered a brisk intracellular $Ca^{2+}$ response. This observation is consistent with the finding that α/β-TCR mAb eliminates a subset of immature thymocytes in fetal thymic organ cultures (Finkel, Marrack et al., 1988). Presumably immature cells that express a functionally coupled CD3/TCR complex undergo negative selection via a clonal deletion mechanism following TCR triggering. If so, one would predict that the 829M and 7-829M cell lines will undergo apoptosis in response to triggering with either cross-linked CD3 or α/β-TCR mAb. Indeed, in transgenic mouse strains expressing class I restricted TCRs, clonal deletion appears to be operative at or prior to the DP maturation stage (Kisielow et al., 1988 and Sha et al., 1988). Thus, it is likely that cells in the immature $CD4^-8^+$ compartment are subject to negative (and perhaps positive) selection processes.

Previous studies have not directly addressed the issue of transcriptional or translational control of CD4 expression in immature $CD4^-8^+$ cells. The Northern blot data in this report demonstrate that CD4 transcripts are not detected in the 829M cell line until conversion to the DP stage after i.t. transfer. Another group suggested that all immature $CD4^-8^+$ cells express CD4 molecules at a density below the level of detection in standard cytofluorometric assays (Nikolic-Zugic et al., 1989). It is conceivable that the $CD4^-8^+$ lymphoma lines described in this report constitutively express CD4 transcripts and surface protein at undetectably low levels. If so, the appearance of CD4 after i.t. transfer may be due to up-regulation of expression rather than de novo synthesis. In either case, the present data suggest that failure to express detectable CD4 antigen on the immature $CD4^-8^+$ subset is regulated at the transcriptional level. Further studies involving nuclear run-on experiments are necessary to determine if the lack (or very low levels) of steady-state CD4 transcripts in $CD4^-8^+$ cells reflect a block in transcription as opposed to alterations in CD4 mRNA processing or stability.

The MNU-induced lymphoma cell lines appear to be reversibly blocked at the immature $CD4^-8^+$ differentiation stage. It is not clear why primary MNU-induced lymphomas and derived cell lines fail to express CD4. MNU is cytotoxic and results in profound depletion of the thymic cortex. However, the differentiation-inducing microenvironment of the thymus appears to remain intact since normal cellularity and CD4/8 subset distribution are restored within 2 weeks after treatment (Stettner et al., in press). Nevertheless, MNU may induce subtle changes in thymic infrastructure that could restrict the developmental program of $CD4^-8^+$ neoplastic cells, which retain the ability to acquire CD4 expression when removed from the modified thymic microenvironment. Perhaps an MNU-induced mutation generates a unique intrathymic peptide that activates transformed thymocytes via the CD3/TCR complex. Activation, in conjunction with a deficiency in appropriate maturation stimuli, may block the cells at the immature $CD4^-8^+$ stage of differentiation. Although speculative, this notion is supported by the recent report that TCR cross-linking inhibits CD4 acquisition by the immature $CD4^-8^+$ normal thymocyte subset (Hunig, 1988). In any event, the cell lines described in this investigation provide useful monoclonal models for further investigations of the immature $CD4^-8^+$ stage of thymocyte differentiation.

### MATERIALS AND METHODS

### Establishment of T-Lymphoma Lines

AKR/J mice were purchased from the Jackson Laboratory (Bar Harbor, ME). AKR/C mice were purchased from Cumberland View Farms (Clinton, TN). Thymic lymphomas were induced by a single i.p. injection of 75 mg/kg of MNU into 4–6-week-old AKR/J mice. Cell lines were established in DMEM (Flow Laboratories, McLean, VA) containing 10% fetal calf serum (FCS) (Hy-Clone Laboratories, Logan, UT) and supplemented with $4\times10^5$ M 2-ME, 4 mM L-glutamine, 1× nonessential amino acids, and 1 mM sodium pyruvate. Parent cell lines were cloned and subcloned by limiting dilution in 96-well microtiter plates.

### Antibodies

Hybridomas 53-6.7 (anti-CD8) and 53-7.3 (anti-CD5) were obtained from Dr. J. Ledbetter (Ledbetter and Herzenberg, 1979). Hybridoma GK 1.5 (anti-CD4) (Dialynas et al., 1983) was obtained from Dr. F. W. Fitch. The J11d hybridoma-producing mAb that recognizes an epitope of HSA was obtained from Dr. M. Bevan (Crispe and Bevan, 1987). A hamster mAb, 500A2, specific for the ε chain of murine CD3 was provided by Dr. J.P. Allison (Allison, et al., 1987). MAb H57-157 detecting a framework determinant of the α/β-TCR was obtained from Dr. R. Kubo (Kubo et al., 1989). Second-step reagents included affinity purified, fluorescein isothiocynate (FITC) conjugated mouse antirat immunoglobulin Ig (Jackson ImmunoResearch, Avondale, PA) and goat antihamster Ig (Cooper Biomedical, Inc., Malvern, PA). FITC conjugated anti-Thy 1.1 (clone TN-26) and anti-Thy 1.2 (clone TS) were purchased from Sigma Chemical Co. (St. Louis, MO). For two-color staining, FITC conjugated anti-CD8 (clone 53-6.7) and phycoerythrin (PE) conjugated anti-CD4 (clone GK 1.5) were purchased from Becton Dickinson (Mountain View, CA). Primary antibodies were airfuged to remove aggregates.

### Immunofluorescence Staining and Flow Cytometry

Single- and two-color staining were performed as previously described. Immunofluorescence was analyzed on a FACS IV (Becton-Dickinson, Mountain View, CA) using a 488-nm argon ion laser. Dead cells were excluded from analysis by forward light scatter. Data were collected using a three-decade log amplifier, stored in the list mode of a Consort 40 PDP/11 based computer. For two-color analysis, contour plots were generated with quadrants based on the profile of unstained cells and cells stained with a single mAb.

### Intrathymic Transfers

Irradiated (1100R) AKR/C mice were reconstituted within 24 hr with $5\times10^6$ AKR/C bone marrow cells injected i.v. After anesthesia, the manubrium and one or two sternabrae were incised and retracted laterally to expose the thymus. Ten µl of lymphoma cells ($10^8$/ml) were infused with a 27-gauge needle into the most accessible lobe. The skin was closed with 6-0 Surgilene (American Cyanamide Co., Danbury, CT) and the mice were allowed to recover in a warm cage.

### RNA Extraction and Northern Blot Analysis

Total RNA was isolated as described (Maniatis et al., 1982) from $10^8$ cells. Poly(A)$^+$ RNA was isolated using the Fast Track Kit from Invitrogen (San Diego, CA). RNA was electrophoresed in 1.1% agarose containing 2.2 M formaldehyde in morpholinopropanesulfonic acid-acetate-EDTA buffer for 420 volt-hr. After transfer to Magna Nylon (MSI, Westborough, MA), blots were hybridized with a nick-translated probe using Denhardt's reagent and formamide (Wahl et al., 1979), washed (MSI Technical Bulletin 103), and analyzed by autoradiography using Kodak X-OMAT AR film and an intensifying screen at −70°C.

### DNA Extraction and Southern Blot Analysis

DNA was extracted as previously described (Hoopes and McClure, 1981), digested with Hind III, and electrophoresed on 0.6% or 0.7% horizontal agarose gels at room temperature for 560 volt-hr in Tris-acetate-EDTA buffer. After transfer to Magna Nylon-66 (MSI, Westborough, MA), the blots were prehybridized and hybridized with a nick-translated probe at 65°C in 5×SSPE, 5× Denhardt's reagent, and 0.5% SDS. The blots were washed and analyzed by autoradiography as described before.

### cDNA Probes

The CD4 probe consists of a 2.4-kb insert of cDNA clone p3C isolated from a thymocyte cDNA library prepared in λgt10 (Littman and Gettner, 1987). The murine Jβ2 probe is a 2.3-kb *Eco*RI fragment of cosmid clone 2.3 W7 subcloned in pUC8 and hybridizes to the entire Jβ2 gene-segment cluster (Malissen et al., 1984).

### Calcium Measurements

Cells were loaded with 1 µM indo-1/AM (Molecular Probes, Eugene, OR) in RPMI 1640 (1% FCS) for 30 min at 37°C, gently pelleted and resuspended in an equal volume of HBSS (1% FCS), and kept at room temperature. Fluorescence measurements were obtained on an Alpha Scan fluorometer (Photon Technology Inc., Princeton, NJ) equipped with dual emission detectors. Background fluorescence was determined from an aliquot of unloaded cells processed in parallel with the loaded cells at densities of $0.5–1\times10^6$ cells/ml. Prior to data acquisition, cells were warmed to 37°C in the water-jacketed cuvette holder. At the end of each recording, digitonin was added to a final concentration of 100 µM to obtain $R_{max}$ and then EGTA/Tris to obtain $R_{min}$. The intracellular free-calcium concentration ($[Ca^{2+}])_i$) was calculated from the 404 nm/490 nm ratio (Grynkiewicz et al., 1985) except that $R_{min}$ and $R_{max}$ were modified by a viscosity correction factor of 1.15 (Poenie, 1990).

### ACKNOWLEDGMENTS

We would like to thank Dale Weiss and Dory Murphy for technical assistance with the i.t. injections and Joyce Mayhugh for help in manuscript preparation. This work was supported by Public Health Service Grants CA-37912 and CA-50604 and by a grant from the Bruce McMillan, Jr. Foundation.

*(Received December 13, 1990)*

*(Accepted January 18, 1991)*

### REFERENCES

Adkins B., Mueller C., Okada C., Reichert R., Weissman I., and Spangrude G. (1986). Early events in T-cell maturation. Annu. Rev. Immunol. **5:** 325–365.

Allison J.P., Havran W., Poenie M., Kimura J., Degraffenreid L., Hamai S., Duve G., Weiss A., and Tsien R. (1987). *In:* The T Cell Receptor, UCLA Symposia on Molecular Biology, New Series, 73rd ed., Kappler J., and Davis M., Eds. (New York: Liss), pp. 33–37.

Ceredig R. (1988). Differentiation potential of 14-day fetal mouse thymocytes in organ culture. Analysis of CD4/CD8-defined single-positive and double-negative cells. J. Immunol. **141:** 355–362.

Ceredig R., Glasebrook A.L., and MacDonald H.R. (1982). Phenotypic and functional properties of murine thymocytes. I. Precursors of cytolytic T-lymphocytes and IL-2 producing cells are all contained within a subpopulation of "mature" thymocytes as analyzed by monoclonal antibodies and flow microfluorometry. J. Exp. Med. **155:** 358–379.

Crispe I.N., and Bevan M.J. (1987). Expression and functional significance of the J11d marker on mouse thymocytes. J. Immunol. **138:** 2013–2018.

Dialynas D.P., Quan Z.S., Wall K.A., Pierres A., Quintans J., Loken M.R., Pierres M., and Fitch F.W. (1983). Characterization of the murine T cell surface molecule, designated L3T4, identified by monoclonal antibody GK 1.5: Similarity of L3T4 to the human Leu3/T4 molecule. J. Immunol. **131:** 2445–2451.

Finkel T.H., Cambier J.C., Kubo R.T., Born W.K., Marrack P., and Kappler J. (1989). The thymus has two functionally distinct populations of immature $\alpha/\beta^+$ T cells: One population is deleted by ligation of $\alpha/\beta$TCR. Cell **58:** 1047–1054.

Finkel T.H., Marrack P., Kappler J., Kubo R.T., and Cambier J.C. (1989). $\alpha/\beta$ T cell receptor and CD3 transduce different signals in immature T cells. Implications for selection and tolerance. J. Immunol. **142:** 3006–3012.

Fowlkes B.J., Edison L., Mathieson B.J., and Chused T. (1985). Early T lymphocytes: Differentiation in vivo of adult intrathymic precursor cells. J. Exp. Med. **162:** 802–822.

Grynkiewicz G., Poenie M., and Tsien R.Y. (1985). A new generation of fluorescence $Ca^{2+}$ indicators with greatly improved fluorescence properties. J. Biol. Chem. **260:** 3440–3450.

Guidos J., Weissman I.L., and Adkins B. (1989). Intrathymic maturation of murine T lymphocytes from $CD8^+$ precursors. Proc. Natl. Acad. Sci. USA **86:** 7542–7546.

Havran W.L., Poenie M., Kimura J., Tsien R., Weiss A., and Allison J.P. (1987). Expression and function of the CD3-antigen receptor on murine $CD4^+8^+$ thymocytes. Nature **330:** 170–173.

Hoopes B.C., and McClure W.R. (1981). Studies on the selectivity of DNA precipitation by spermine. Nucleic Acids Res. **9:** 5493–5504.

Hunig T. (1988). Cross-linking of the T cell antigen receptor interferes with the generation of $CD4^+8^+$ thymocytes from their immediate $CD4^-8^+$ precursors. Eur. J. Immunol. **18:** 2089–2092.

Kisielow P., Bluthmann H., Staerz U.D., Steinmetz M., and von Boehmer H. (1988). Tolerance in T-cell-receptor transgenic mice involves deletion of nonmature $CD4^+8^+$ thymocytes. Nature **333:** 742–746.

Kubo R.T., Born W.T., Kappler J.W., Marrack P., and Pigeon M. (1989). Characterization of a monoclonal antibody which detects all murine $\alpha/\beta$ T cell receptors. J. Immunol. **142:** 2736–2742.

Ledbetter J.A., and Herzenberg L.A. (1979). Xenogeneic monoclonal antibodies to mouse lymphopid differentiation antigens. Immunol. Rev. **47:** 63–90.

Littman D., and Gettner S.N. (1987). Unusual intron in the immunoglobulin domain of the newly isolated murine CD4 (L3T4) gene. Nature **325:** 453–455.

MacDonald H.R., Budd R.C., and Howe R.C. (1988). A $CD3^-$ subset of $CD4^-8^+$ thymocytes: A rapidly cycling intermediate in the generation of $CD4^+8^+$ cells. Eur. J. Immunol. **18:** 519–523.

McPhee D., Pye J., and Shortman K. (1979). The differentiation of T lymphocytes. V. Evidence for intrathymic death of most thymocytes. Thymus **1:** 151–162.

Malissen M., Minard K., Mjolsness S., Kronenberg M., Goverman J., Hunkapiller T., Prystowsky M.B., Yoshikai Y., Fitch F., Mak T.W., and Hood L. (1984). Mouse T cell antigen receptor: Structure and organization of constant and joining gene segments encoding the $\beta$ polypeptide. Cell **37:** 1101–1110.

Maniatis T., Fritsch E.F., and Sambrook J. (1982). *In:* Molecular Cloning (Cold Spring Harbor, NY: Cold Spring Harbor Laboratories), pp. 191–192.

Nikolic-Zugic J., and Bevan M.J. (1988). Thymocytes expressing CD8 differentiate into $CD4^+$ cells following intrathymic injection. Proc. Natl. Acad. Sci. USA **85:** 8633–8637.

Nikolic-Zugic J., Moore M.W., and Bevan M.J. (1989). Characterization of the subset of immature thymocytes which can undergo rapid in vitro differentiation. Eur. J. Immunol. **19:** 649–653.

Penit C., and Vasseur F. (1988). Sequential events in thymocyte differentiation and thymus regeneration revealed by a combination of bromodeoxyuridine DNA labeling and antimitotic drug treatment. J. Immunol. **140:** 3315–3323.

Poenie M. (1990). Alteration of intracellular fura-2 fluorescence by viscosity: A simple correction. Cell Calcium **11:** 85–91.

Richie E.R. (1988). N-methyl-N-nitrosourea and spontaneous AKR/J thymic lymphomas express distinct differentiation antigen phenotypes. Leuk. Res. **12:** 233–242.

Richie E.R., McEntire B., Crispe N., Kimura J., Lanier L.L., and Allison J.P. (1988). $\alpha/\beta$ T-cell antigen receptor gene and protein expression occurs at early stages of thymocyte differentiation. Proc. Natl. Acad. Sci. USA **85:** 1174–1178.

Scollay R., Bartlett P., and Shortman K. (1984). T-cell development in the adult murine thymus: Changes in the expression of the surface antigens Ly2, L3T4 and B2A2 during development from early precursor cells to emigrants. Immunol. Rev. **82:** 79.

Sha W.C., Nelson C.A., Newberry R.D., Kranz D.M., Russel J.H., and Loh D.Y. (1988). Positive and negative selection of an antigen receptor on T cells in transgenic mice. Nature **336:** 73–76.

Shortman K., Wilson A., Egerton M., Pearse M., and Scollay R. (1988). Immature $CD4^-CD8^+$ murine thymocytes. Cell. Immunol. **113:** 462–479.

Stettner S.L., Rummel S.A., Conti C.J., and Richie E.R. (in press). N-methyl-N-nitrosourea alters thymocyte subset distribution and targets immature CD4$^{-}$8$^{+}$ cells for lymphoma development. Cancer Res.

Wahl G.M., Stern M., and Stark G.R. (1979). Efficient transfer of large DNA fragments from agarose gels to diazobenzyloxymethyl-paper and rapid hybridization using dextran sulfate. Proc. Natl. Acad. Sci. USA **76:** 3683–3687.

Yotoku M., Grossberg A.L., Stout R., and Herzenberg L.A. (1976). Further studies on ThB, a cell surface antigenic determinant present on mouse B cells, plasma cells and immature thymocytes. Cell. Immunol. **23:** 140–157.

